# South Shields Guardians and the Use of Alcohol

**Published:** 1914-02-14

**Authors:** Thomas Dutton

**Affiliations:** 25 New Cavendish Street, W.


### South Shields Guardians and the Use of Alcohol.

*To the Editor of* The Hospital

Sir,—I was very pleased to read the remarks on the above subject in The Hospital, January 31, p. 469, which I cordially endorse. For a Board of Guardians to take autocratic power and dictate to a skilled medical officer as to the use of or quantity of alcohol in the treatment of disease is impudent in the extreme, and I should think *ultra vires*.

Alcohol is just as necessary a drug to keep in stock as opium, quinine, or cocaine. We all know the serious harm excess of alcohol has on the health and morals of a community, so also we know equally that opium in excess does more serious and lasting damage. No sane body of men would think of not allowing the use of opium because of its abuse. I am sorry to know the abuse of opium is far greater than is generally believed among the lay public.—I am, Sir, yours truly,

Thomas Dutton (M.D., M.R.C.P.).

25 New Cavendish Street, W.
February 4, 1914.

